# Operation decision of competitive mining supply chain based on social responsibility

**DOI:** 10.1371/journal.pone.0278815

**Published:** 2022-12-08

**Authors:** Wenyi Du, Huimin Wang

**Affiliations:** 1 Business School, Hohai University, Nanjing, China; 2 Business School, Jiangsu Normal University, Xuzhou, China; Shanghai Ocean University, CHINA

## Abstract

The development of the green economy has significantly impact the traditional mining industry. Mining enterprises must invest in green technology to reduce the environmental pollution caused by flying dust and soil erosion and are subject to increased scrutiny to be socially responsible when conducting their business. To address this issue, we consider a competitive mining supply chain system consisting of two excavators and two exclusive retailers. Among them, the excavators have a certain sense of corporate social responsibility (CSR), that is, in addition to pursuing economic profits, they also consciously pay attention to the interests of consumers. We establish three different game models that two excavators exhibit no CSR behaviour (NN), two excavators exhibit CSR behaviour (SS) and one excavator exhibits CSR behaviour (SN). We examine the optimal decision-making strategies and analyse the impact of social responsibility. Analytical results show that the optimal strategies of mining supply chain are different under different supply chain structures. The optimal decisions of the mining supply chain members are the same in each case under the *NN* and *SS* models. In the *SN* model, the optimal decision strategy value of mining supply chain members is always greater than non-socially responsible supply chain members. In SS model, when the intensity of social responsibility competition is low, two excavators reduce the wholesale price, and retailers reduce the sales price; when the intensity of social responsibility competition is strong, two excavators will increase the wholesale price, and retailers will increase the sales price. These help to promote product sales and increase the profits of the supply chain system. In SN model, with the increase of social responsibility competition intensity, the wholesale price of two excavators and the sales price of retailers first increased and then decreased. Finally, numerical examples illustrated to justify the proposed model.

## 1 Introduction

In recent years, the sustainable development of the economy, society, and environment has received global attention. Environmental pollution, soil erosion, mining accidents, and other major accidents impacting societies occur frequently [1, 2], such as the “Senghenydd colliery disaster,” the “Bohai Bay oil spill,” and the “Yantai 1.10 mining disaster.” Over time, such incidents have attracted greater attention to the problem of corporate social responsibility (CSR) and sustainable development [3]. Product recycling [4] and recycling of waste products [5] can effectively reduce environmental pollution and resource waste. Increasingly more enterprises are trying to increase social responsibility cost investments, improve production technology, engage in lean production [6], improve product quality [7], and reduce carbon emissions in the production process [8]. Enterprises can improve their corporate image by improving their level of social responsibility [9, 10]. While pursuing the maximization of their own interests, enterprises pay attention to and promote the interests of customers, employees, suppliers, local communities, and other stakeholders [11, 12]. In this way, CSR promotes social harmony, stability and sustainable development.

Sustainable development is the long-term goal of competition for an enterprise. Enterprises have to face the terminal demand correctly. However, the demand for retail terminals is becoming more and more personalized and diversified. For modern enterprises, depending on their own strength, it is difficult to fully meet the market demand and cope with the complex market environment [13, 14]. Enterprises and upstream and downstream enterprises need to establish a stable cooperative operation and symbiotic win-win relationship [15, 16]. In this way, enterprises can fully meet customer needs and enhance competitiveness [17]. However, the essence of market competition is the competition between supply chains and supply chains [18]. CSR behaviour has also begun to expand from individual enterprises to the entire supply chain. A single enterprise failing to incorporate social responsibility into its operations can quickly spread risk to other enterprises in the same chain or competitive supply chain by means of an unfavourable incident such as “unlucky event” [19]. Enterprises that establish CSR can maintain their brand reputation, boost their financial value [20], and increase consumer product demand [21, 22].

Motivated by this observation, we considered the pricing mechanism and social responsibility of the mineral supply chain under the competitive environment, including wholesale price, sales price and social responsibility level. We investigate the impact of competition intensity and social responsibility level on the supply chain operation, and solve how to optimize the pricing and social responsibility level to improve the performance of the mineral supply chain. In this framework, the first thing we construct a competitive mineral supply chain system. Secondly, we establish three game models of social responsibility on different subjects, and solve the optimal strategy of mineral supply chain in each case. Then, we analyse and discuss how the change of parameters affects the decision-making strategies of supply chain members with different social responsibility action. Finally, we verify the impact of parameter changes on supply chain performance through numerical analysis.

The rest of this paper structured as follows. Section 2 is a literature review, followed by Section 3, which describes the problem and symbols. Section 4 explains the decision-making solution and provides an analysis of the competitive supply chain in three situations. Section 5 is a sensitivity analysis that observes how the competitive intensity of social responsibility affects the optimal decision-making of supply chain members. Finally, Section 6 summarizes and concludes the paper.

## 2 Literature review

This paper mainly carries out the literature review from two aspects: one is the supply chain decision-making of social responsibility and the other is the operation of competitive supply chain. Next, we will start the literature review along these two routes.

### 2.1 Supply chain decision-making of social responsibility

In the research on supply chains, some studies have begun to focus on the problem of CSR. Some scholars discuss the supply chain decision-making problem with CSR from the perspectives of consumer utility [23], cost information asymmetry [24], CSR effort level [25, 26]. The paper studied the social problems in the supply chain through case studies and discussed in detail how managers can work actively to reduce social risks, create new opportunities, and improve company performance [27]. In the remanufacturing supply chain, considering the differences of corporate social responsibility and consumers’ payment for new products and remanufactured products, discusses their impact on the decisions and profits of the remanufacturing supply chain members [28, 29]. The sharing mechanism of social responsibility investment can also improve the performance of the supply chain [30, 31]. The social responsibility input behavior of different entities will affect the price, profit and social responsibility level of the closed-loop supply chain [32], while the choice of different sales channels and different dominant modes will affect the optimal decision-making and social responsibility level of supply chain members [33]. Different government subsidy strategies can improve the performance of social responsibility supply chain [34, 35]. The impacts of CSR investment and government subsidies on new product pricing and waste recycling is analysed in the closed-loop supply chain [36–38].

Extant researches show that the level of social responsibility affects the decision-making of supply chain members and systems, even their profits. Nevertheless, little attention are paid to operate the competitive supply chain with different levels of social responsibility. When there is competition, the production practice of enterprises can choose different levels of social responsibility, which in turn affects the decision-making optimization and performance of competitive supply chains.

### 2.2 Operation of competitive supply chain

The first proposed the competitive supply chain came from the field of marketing research. Mcguier and Staelin analysed the dominant vertical structure of two manufacturers and two exclusive retailers for a certain linear demand function and found that the decentralized structure enables manufacturers strategically to avoid severe price competition [39]. Later, scholars expanded the research on the competitive supply chain. Risk aversion coefficient, demand uncertainty factors, and service investment efficiency affect the optimal decisions of supply chain members [40, 41]. Over time, the intensity of competition between chains increases [42, 43]. A supply chain with high supply reliability has higher order quantity and a stronger competitive advantage [44, 45]. An investigation of supply chain cooperation under the conditions of sharing and not sharing advertising information found that the sharing of advertising information can improve the overall performance of the supply chain [46, 47]. Once the information entering the market is known by competitors, private brands’ rivals will change their original operation strategies [48, 49]. Meng et al. studied the operation strategy of a personalized product supply chain in two control structures of “manufacturing and marketing separation” and “customization integration” [50]. Compared with the separation structure of manufacturing and marketing, the personalized product supply chain opts for the customized integrated structure [51, 52]. In most cases, after integrating with suppliers, manufacturers will invest more money into supplier development; moreover, both manufacturers will integrate with suppliers in an equilibrium state [53, 54].

Extant researches show that competitive environment affects the decision and performance of supply chain members. Nevertheless, little attention are paid to operate the competitive supply chain considering enterprises’ CSR. In today’s world, enterprises cannot exist from the supply chain. While pursuing the maximization of their own interests, enterprises also need to pay attention to promoting the interests of customers, employees, suppliers, local communities, and other stakeholders. This is the embodiment of CSR, which can promote social stability and sustainable economic development.

Most of these studies analysed the single supply chain decision-making and performance in the social responsibility scenario, and the supply chain decision-making and performance in the competitive environment. This research takes different perspectives to determine the optimal decision and performance. It is aiming to observe how different entities operate the competitive supply chain in the context of social responsibility, and how changes in parameters affect the strategy and performance of mineral supply chain members.

In summary, our study contributes to the literature in three aspects. Firstly, we consider the competitive supply chain operation decision-making problem under the CSR scenario, which makes up for the gap in the single chain supply chain social responsibility research. Secondly, we derive three game models of competitive supply chains, and provide the optimal decisions of members in each case. Finally, we examine how CSR-related competition affects the operation decisions of the members of a competitive mining supply chain and explicate the evolution path of the operation decisions of the members of the mining supply chain in three game situations with the help of the example analysis.

## 3 Model description

**Fig 1:**
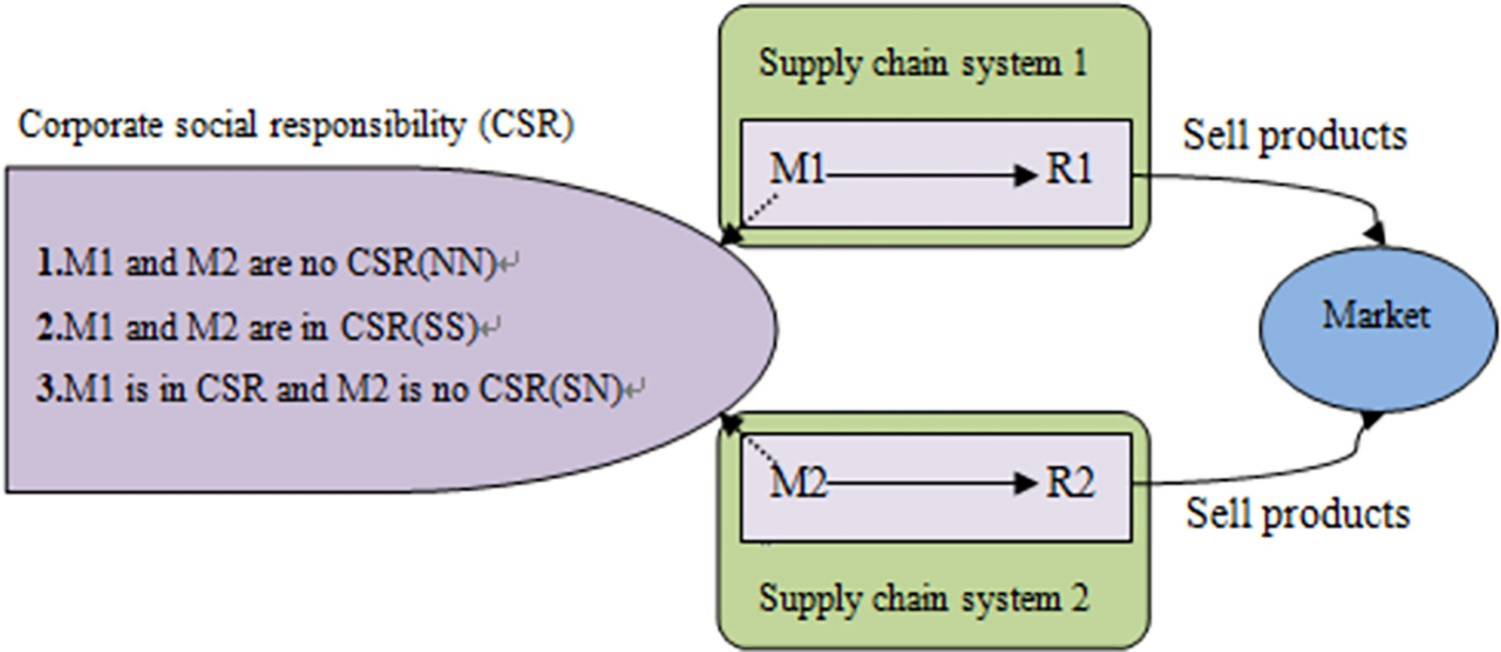
Flowchart of the competitive supply chain system with CSR.

In this paper, we consider a competitive supply chain system consisting of two excavators (M1 and M2) and two exclusive retailers (R1 and R2). In Fig 1, the solid arrows show the product flow/transfer. M1 and M2 represent the two excavators from the two routes, and R1 and R2 represent the two retailers at the same location.

The excavator can have a certain sense of social responsibility, i.e., in addition to pursuing economic profits, they also consciously pay attention to the environmental and the health of local people (e.g., green production of better and more environmentally-friendly products). According to the assumption in the literature [26], retailers only need to do a good job in sales. The retailers have no social responsibility. In each supply chain, the excavator is the leader and first determines the wholesale price, after which the two retailers decide on their own prices. Basis of [12] research and the theory of competitive supply chain, the following equation can be derived:

$$q_{i}=a-p_{i}+bp_{3-i}+\eta_{i}-k\eta_{3-i}\;(i=1,2)$$ (1)

where *q*_*i*_ is the market sales volume of retailer *i*. *a* is the maximum demand of products in the market. *p*_*i*_,*p*_3−*i*_ are the market sales prices of retailers *i* and 3*-i*. *b* is the price sensitivity coefficient (price competition intensity or competition intensity among retailers), *η*_*i*_,*η*_3−*i*_ are the social responsibility level of excavators *i* and 3*-i*, and *k* is the social responsibility sensitivity coefficient (social responsibility competition intensity), *k*≥0.

Combined with literature [24], the social responsibility cost input function is

$$c_{i}=\frac{1}{2}\eta_{i}^{2}$$ (2)

where the social responsibility level of excavator *i* is *η*_*i*_, and *c*_*i*_ is the social responsibility input cost of excavator *i*.

## 4 Decision analysis

### 4.1 Two excavators exhibit no CSR behaviour (NN)

Both excavators do not consider their own CSR behaviour and have no social responsibility cost input. The excavator first decides the wholesale price, and then the retailers decide the order quantity. At this time, the expected profit of each member in the supply chain system can be expressed as follows:

$$M_{i}^{NN}=w_{i}^{NN}q_{i}^{NN}$$ (3)

$$R_{i}^{NN}=(p_{i}^{NN}-w_{i}^{NN})q_{i}^{NN}$$ (4)

**Proposition 1** *When there is no social responsibility behaviour in the supply chain system, the excavators’ optimal wholesale price and the retailers’ optimal order quantity are $w_{i}^{NN*}$ and $q_{i}^{NN*}$, respectively (See S1 Appendix).*

Proposition 1 indicates that if the enterprise exhibits no social responsibility behaviour, it will completely adhere to the competitive supply chain operation strategy, and the excavator and retailer can obtain the optimal decision-making strategy.

### 4.2 Two excavators exhibit CSR behaviour (SS)

Both excavators undertake their own CSR behaviour in the process of product production, which requires a certain amount of social responsibility cost input. The excavator should not only decide the wholesale price but also the level of social responsibility (the level of social responsibility determines the amount of social responsibility capital investment), and the retailer should decide the order quantity.

At this time, the expected profit of each member in the supply chain system can be expressed as follows:

$$M_{i}^{SS}=w_{i}^{SS}q_{i}^{SS}-\frac{1}{2}\eta_{i}^{SS2}$$ (5)

$$R_{i}^{SS}=(p_{i}^{SS}-w_{i}^{SS})q_{i}^{SS}$$ (6)

**Proposition 2** *The optimal wholesale price and the optimal social responsibility level of the excavators are $w_{i}^{SS*}$ and $\eta_{i}^{SS*}$, respectively, and the optimal order quantity of the retailers is $q_{i}^{SS*}$ (See S1 Appendix).*

Proposition 2 shows that when excavators undertake CSR, the optimal wholesale price and optimal social responsibility level of excavators in different chains will be the same, and retailers will also choose the same order quantity. At this time, the same role members in different chains will choose the same policy, which is slightly beneficial to their profit maximization.

### 4.3 One excavator exhibits CSR behaviour (SN)

In the two chains, only one chain excavator shows CSR behaviour. The paper may assume that the excavator in the first chain exhibits social responsibility behaviour, while the excavator in the second chain does not. In this case, Excavator 1 decides not only the wholesale price but also the level of social responsibility (the level of social responsibility determines the capital investment of social responsibility). In contrast, Excavator 2 only decides the wholesale price, and the retailer then decides the order quantity.

At this time, the expected profit of each member can expressed as follows:

$$M_{1}^{SN}=w_{1}^{SN}q_{1}^{SN}-\frac{1}{2}\eta_{1}^{SN2}$$ (7)

$$M_{2}^{SN}=w_{2}^{SN}q_{2}^{SN}$$ (8)

$$R_{i}^{SN}=(p_{i}^{SN}-w_{i}^{SN})q_{i}^{SN}$$ (9)

**Proposition 3** *When the excavator of only one of the two chains exhibits CSR behaviour, the optimal wholesale price and social responsibility level of Excavator 1 are $w_{1}^{SN*}$ and $\eta_{1}^{SN*}$, the optimal wholesale price of Excavator 2 is $w_{2}^{SN*}$, and the optimal order quantity of Retailer 1 and Retailer 2 are $q_{1}^{SN*}$ and $q_{2}^{SN*}$, respectively (See S1 Appendix).*

Proposition 3 shows that in the competitive supply chain system, only one excavator in the chain undertakes social responsibility, while the other does not. In this case, the optimal decision strategies of the members (excavator and retailer) in the two chains differ. The supply chain decision-making strategy of the excavator undertaking social responsibility is improved, which is always greater than that of the excavator without social responsibility. This further shows that CSR behaviour is conducive not only to the decision-making strategy of its enterprise but also to the improvement of the decision-making strategy of other enterprises in the supply chain.

## 5 Sensitivity analysis

**Proposition 4** *In the case that both supply chains exhibit a sense of social responsibility, when* $k^{o}>k>\frac{\left(4+b)(2-b\right)}{4b}$, then $\frac{\partial q_{i}^{SS}}{\partial k}>0$, $\frac{\partial w_{i}^{SS}}{\partial k}>0$; when $0\leq k\leq \frac{\left(4+b)(2-b\right)}{4b}$, then $\frac{\partial q_{i}^{SS}}{\partial k}<0$, $\frac{\partial w_{i}^{SS}}{\partial k}<0$. *k is the upper threshold of social responsibility (See S1 Appendix).*

Proposition 4 shows that the excavators in both chains bear social responsibility. When the competition intensity of social responsibility is high, the wholesale price of the excavators and the order price of the retailers are both decreasing functions of the competitive intensity of social responsibility. This is mainly because at the beginning of social responsibility, the competition among excavators is relatively small, and each excavator needs to consider numerous factors. The initial cost investment is large, which is not conducive to the improvement of decision-making strategies of excavators and retailers. With the increase of social responsibility competition intensity among excavators, when the social responsibility competition intensity is at $\left(\frac{\left(4+b)(2-b\right)}{4b},k^{0}\right)$, the wholesale price of the excavator and the order price of the retailer are both increasing functions of social responsibility competition intensity. At this time, the wholesale price of the excavator is larger. However, the retailer’s order quantity is also higher, which contrasts with the general economic principle of “high price, lower quantity.” This is mainly due to consumers’ pursuit of products produced by socially responsible enterprises, which is grounded in the belief that the products produced by enterprises that exhibit social responsibility are relatively good. In the contemporary market, more and more consumers are happy to pay higher prices to buy green products.

The management enlightenment of Proposition 4 is as follows: In a competitive environment, when two excavators assume social responsibility, both sides of the competition will increase the product price, that is, the social responsibility behavior intensifies the price competition. It further shows that social responsibility behavior needs cost input.

**Proposition 5** *In the condition that both supply chains have a sense of social responsibility, when $0<k<k^{o}$, then* $\frac{\partial \eta_{i}^{SS}}{\partial k}>0$ *(See S1 Appendix).*

Proposition 5 shows that excavators in both chains exhibit social responsibility, and the greater the social responsibility competition among excavators, the higher the level of CSR. The main reason is that once an excavator has a sense of social responsibility, it will produce better green products. Although the price is high, it will still win the favour and trust of consumers, leading to purchase behaviour. In the market, the products of enterprises with a strong sense of CSR have a competitive advantage that causes other excavators to improve their social responsibility level, strengthen social responsibility cost input, and produce products with strong competitiveness, all of which is conducive to the improvement of product quality throughout the market.

The management enlightenment of Proposition 5 is as follows: In a competitive environment, when two excavators undertake social responsibility, the level of social responsibility can improve the green of products, that is, higher quality and less pollution.

**Proposition 6** *In the condition that one of the two supply chains exhibits social responsibility, when $\frac{8-b^{2}}{2b}<k<k^{o}$, then $\frac{\partial q_{1}^{SN}}{\partial k}<0$, $\frac{\partial w_{1}^{SN}}{\partial k}<0$; when $0\leq k\leq \frac{8-b^{2}}{2b}$,then $\frac{\partial q_{1}^{SN}}{\partial k}>0$, $\frac{\partial w_{1}^{SN}}{\partial k}>0$ (See S1 Appendix).*

Proposition 6 shows that only one excavator in the two chains exhibits social responsibility. When the competition intensity of social responsibility is $\left[0,\frac{8-b^{2}}{2b}\right]$, the wholesale price of the excavator and the order price of the retailer in the supply chain are both increasing functions of the competitive intensity of CSR. The wholesale price of the excavator’s products is larger, but the retailer’s order quantity is also higher, which again differs from the general economic principle of “high price, lower quantity.” This is mainly due to consumers’ pursuit of products produced by socially responsible enterprises, which is found in the belief that the products produced by enterprises that exhibit CSR are relatively good. In the market, increasingly more consumers are willing to pay higher prices to purchase green products. Given the increasing competition intensity of social responsibility among excavators, when the competition intensity of social responsibility is $\left(\frac{8-b^{2}}{2b},k^{o}\right)$, the wholesale price of excavators and the order price of retailers in the supply chain are both the decreasing functions of the competitive intensity of CSR. This is mainly due to that the competitors do not bear the level of social responsibility and do not engage in social responsibility cost input. The social responsibility cost of the excavators who bear the social responsibility is relatively high. As the products in the market are mixed, the supply chain without social responsibility takes advantage of the low price to improve its competitiveness. This phenomenon is similar to the concept of “bad money drives good money,” which is not conducive to supply chain social responsibility. However, the decision-making strategies of excavators and retailers are improved. Further analysis shows that the change in competition intensity of social responsibility among excavators will also lead to the decision-making strategies of members in the supply chain who do not undertake social responsibility. See Proposition 7 for details.

**Proposition 7** *In the condition where one of the two supply chains exhibits social responsibility, when $\frac{-b^{5}+20b^{3}-96b+\sqrt{H_{1}}}{8b}<k<k^{o}$,then $\frac{\partial q_{2}^{SN}}{\partial k}<0$, $\frac{\partial w_{2}^{SN}}{\partial k}<0$; when $0\leq k\leq \frac{-b^{5}+20b^{3}-96b+\sqrt{H_{1}}}{8b}$, then $\frac{\partial q_{2}^{SN}}{\partial k}>0$, $\frac{\partial w_{2}^{SN}}{\partial k}>0$. $H_{1}=12288-4608b^{2}+688b^{4}-44b^{6}+b^{8}$ (See S1 Appendix).*

Proposition 7 shows that only one excavator in the two chains bears social responsibility. When the competition intensity of social responsibility is $\left[0,\frac{-b^{5}+20b^{3}-96b+\sqrt{H_{1}}}{8b}\right]$, the wholesale price of the excavators and the order price of the retailers in the supply chain where the social responsibility is not assumed are both increasing functions of the competitive intensity of CSR. The main reason is that consumers do not differentiate between the products produced by socially responsible enterprises and those produced by non-social responsible enterprises. Some consumers will choose the products of enterprises with low levels of social responsibility, and others will choose to purchase the products of non-socially responsible enterprises with lower prices. However, with the increase of social responsibility level, the wholesale price of the excavators and the order price of the retailers in the supply chain where social responsibility is not present are both the decreasing function of the competitive intensity of social responsibility. At this time, there is a significant difference in the green degree of the products produced by the two, and consumers will be more willing to choose the products of the social responsibility enterprises, resulting in poor consumer recognition of the products of non-socially responsible enterprises.

The management enlightenment of Proposition 7 is as follows: In a competitive environment, when an excavator undertakes social responsibility behavior, at the beginning, a lower level of social responsibility behavior will intensify the price competition of products, and greener products are often not accepted by people. In the market, there are many enterprises undertaking non-social responsibilities, and everyone has a herd mentality. With the improvement of social responsibility behavior, consumers will turn to greener products. Because the products produced by enterprises with high social responsibility are of good quality and pollution-free.

**Proposition 8** *In the condition where one of the two supply chains exhibits social responsibility*, *when* 0<*k*<*k*^*o*^, *then* $\frac{\partial \eta_{1}^{SN}}{\partial k}>0$.

Proposition 8 shows that only one excavator in the two chains exhibits social responsibility. The stronger the competition of social responsibility among excavators, the higher the level of CSR, the main reason being the presence of an excavator with a sense of social responsibility that will produce better green products. Although the price is high, such an excavator will still win the favour and trust of consumers, leading to purchase behaviour. Once consumers start to purchase more products from CSR enterprises, they will increase their social responsibility level and produce more green and better products to meet consumer needs in the market.

The management enlightenment of Proposition 4 is as follows: In a competitive environment, when an excavator undertakes social responsibility, the improvement of competitive social responsibility has promoted more consumers to turn to greener products. These green products are of good quality and pollution-free. The products produced by enterprises with high social responsibility have become the main theme of the market, which is conducive to the formation of a healthier and better market mechanism.

## 6 Numerical examples

In order to further analyze the relationship between social responsibility competition intensity and decision variables, we further assume that the maximum demand for products in the market is 100 units, that is, *a* = 100. The competition intensity among retailers can be divided into three situations: weak competition, general competition and strong competition, which are represented by b = 0.2, b = 0.5 and b = 0.8, respectively. The specific relationship diagram shows below.

**Fig 2:**
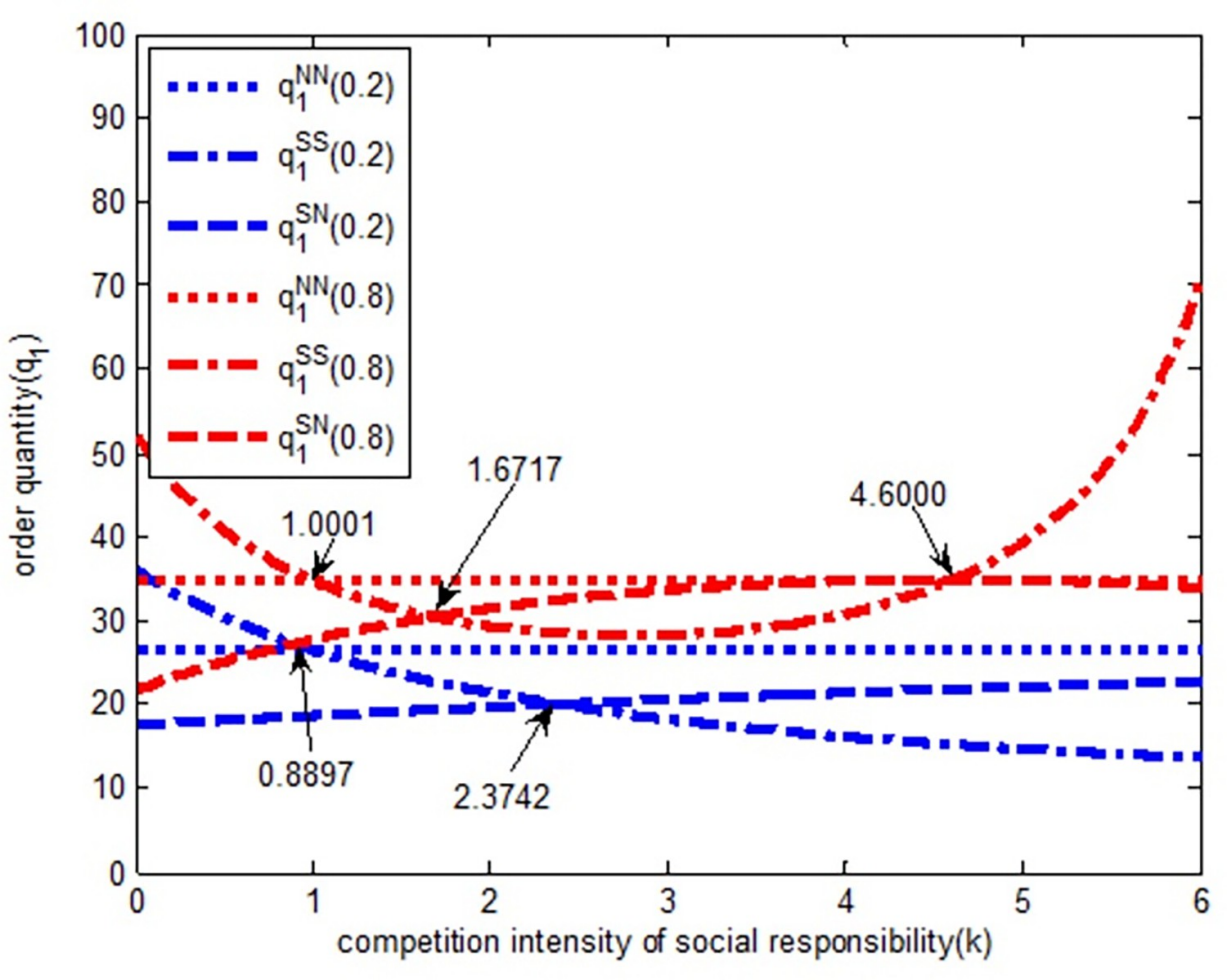
The relationship between order quantity *q*_*1*_ and completion intensity of social responsibility.

As shown in Fig 2, when the quantity competition intensity is weak (blue line in Fig 2), in *SS* model, the order quantity of Retailer 1 is an increasing function of social responsibility competition intensity. In other words, with the increase of social responsibility competition intensity, the order quantity of Retailer 1 gradually increases. In SN model, the order quantity of Retailer 1 is a decreasing function of social responsibility competition intensity, i.e., with the increase of responsibility competition intensity, the order quantity of Retailer 1 decreases gradually. When the competition intensity of social responsibility is (0, 0.8897), the order quantity relationship of Retailer 1 is $q_{1}^{SS}>q_{1}^{NN}>q_{1}^{SN}$. Retailer 1’s order quantity evolves gradually according to the *SS→NN→SN* model; when the social responsibility competition intensity is (0.8897, 2.3742), the order quantity relationship of Retailer 1 under the three models is $q_{1}^{NN}>q_{1}^{SS}>q_{1}^{SN}$. When the social responsibility competition intensity is (2.3742, 6), the order quantity of Retailer 1 under the three models is $q_{1}^{NN}>q_{1}^{SN}>q_{1}^{SS}$, and it evolves gradually according to *NN→SN→SS* model.

When the quantity competition intensity is strong (red line in Fig 2) in *SS* model, the order quantity of Retailer 1 first decreases and then increases with the increase in social responsibility competition intensity. Likewise, in SN model, the order quantity of Retailer 1 first increases and then decreases with the increase of social responsibility competition intensity. However, with the increase in social responsibility competition intensity, the quantity competition is strong. Most of the order quantity with a strong degree of competition is larger than that with weak quantity competition, that is, most of the red line is above the black line. Therefore, in a competitive environment, CSR is conducive to the increase of Retailer 1’s order quantity. When the social responsibility competition intensity is (0, 1.0001), under the three models, the order quantity relationship of Retailer 1 is $q_{1}^{SS}>q_{1}^{NN}>q_{1}^{SN}$, and it evolves gradually according to the *SS→NN→SN* model. When the social responsibility competition intensity is (1.0001, 1.6717), the order quantity relationship of Retailer 1 under the three models is $q_{1}^{NN}>q_{1}^{SS}>q_{1}^{SN}$, and it evolves gradually according to the *NN→SS→SN* model. When the social responsibility competition intensity is (1.6717, 4.6000), the order quantity relationship of Retailer 1 under the three models is $q_{1}^{NN}>q_{1}^{SN}>q_{1}^{SS}$, and it evolves gradually according to the *NN→SN→SS* model. Finally, when the social responsibility competition intensity is (4.6000, 6), the order quantity relationship of Retailer 1 under the three models is $q_{1}^{SS}>q_{1}^{NN}>q_{1}^{SN}$, and it evolves gradually according to the pattern of *SS→NN→SN*.

**Fig 3:**
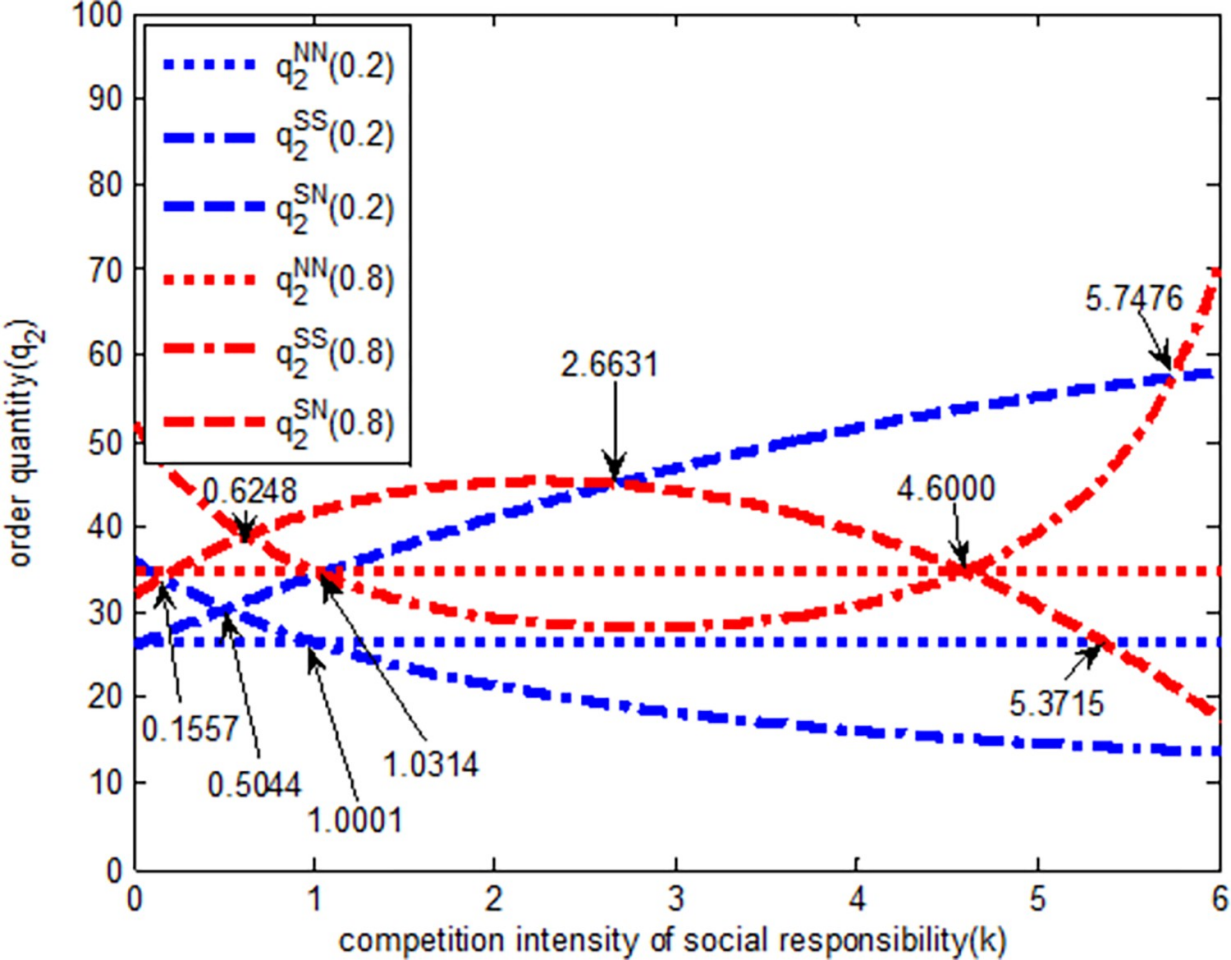
The relationship between order quantity *q*_*2*_ and completion intensity of social responsibility.

As shown in Fig 3, when the quantity competition intensity is weak (blue line in the Fig 3), in *SS* model, the order quantity of Retailer 2 is a decreasing function of social responsibility competition intensity. In other words, with the increase of social responsibility competition intensity, the order quantity of Retailer 2 gradually decreases. In SN model, the order quantity of Retailer 2 is an increasing function of social responsibility competition intensity, i.e., with the increase of responsibility competition intensity, the order quantity of Retailer 2 increases gradually. When the competition intensity of social responsibility is (0, 0.5044), the order quantity relationship of Retailer 2 is $q_{2}^{SS}>q_{2}^{SN}>q_{2}^{NN}$. Retailer 2’s order quantity evolves gradually according to the *SS→SN→NN* model; when the social responsibility competition intensity is in (0.5044, 1.0001), the order quantity relationship of Retailer 2 under the three models is $q_{2}^{SN}>q_{2}^{SS}>q_{2}^{NN}$. It evolves gradually according to the *SN→SS→NN* model. When the social responsibility competition is (1.0001, 6), the order quantity relationship of Retailer 2 is $q_{2}^{SN}>q_{2}^{NN}>q_{2}^{SS}$, and it evolves gradually according to *SN→NN→SS* model.

When the quantity competition intensity is strong (red line in the Fig 3) in *SS* model, the order quantity of Retailer 2 first decreases and then increases with the increase in social responsibility competition intensity. Likewise, in *SN* model, the order quantity of Retailer 2 first increases and then decreases with the increase of social responsibility competition intensity. Therefore, when the social responsibility competition intensity is high, the *SS* model is conducive to increasing retailer 2’s order quantity. When the social responsibility competition intensity is in (0, 0.6248), under the three models, the order quantity relationship of Retailer 2 is $q_{2}^{SS}>q_{2}^{SN}>q_{2}^{NN}$, and it evolves gradually according to the *SS→SN→NN* model. When the social responsibility competition intensity is (0.6248, 1.0314), the order quantity relationship of Retailer 2 under the three models is $q_{2}^{SN}>q_{2}^{SS}>q_{2}^{NN}$, and it evolves gradually according to the *SS→SN→NN* model. When the social responsibility competition intensity is (1.0314, 4.6000), the order quantity relationship of Retailer 2 under the three models is $q_{2}^{SN}>q_{2}^{NN}>q_{2}^{SS}$ and it evolves gradually according to the *SN→ NN→SS* model. Finally, when the social responsibility competition intensity is (4.6000, 6), the order quantity relationship of Retailer 2 under the three models is $q_{2}^{SS}>q_{2}^{NN}>q_{2}^{SN}$, and it evolves according to the *SS→NN→SN*.

**Fig 4:**
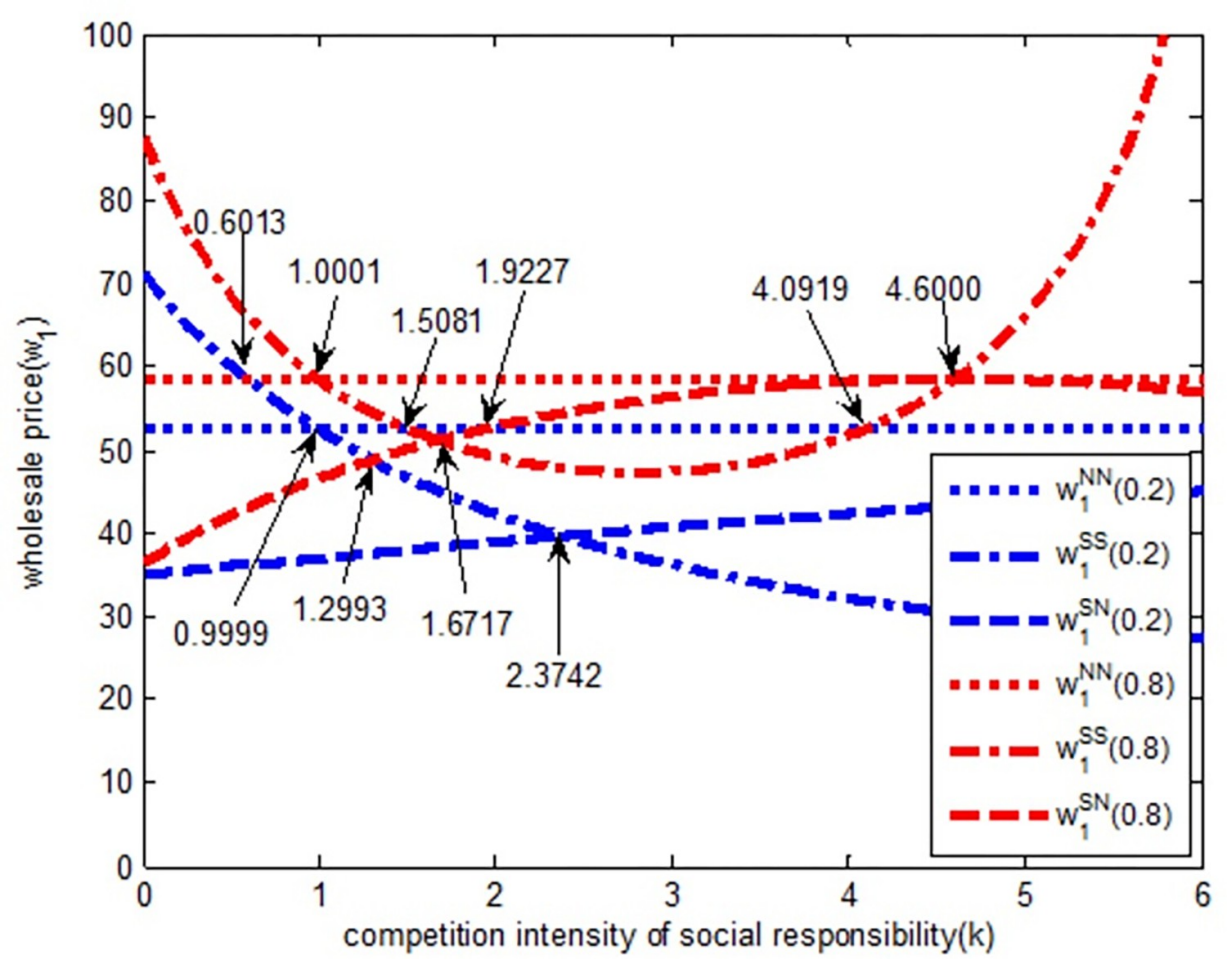
The relationship between wholesale price *w*_*1*_ and completion intensity of social responsibility.

As shown in Fig 4, when the intensity of quantity competition is weak (blue line in Fig 4), in *SS* model, the wholesale price of Excavator 1 is a decreasing function of social responsibility competition intensity. With the increase in social responsibility competition intensity, the wholesale price of Excavator 1 gradually decreases. In *SN* model, the wholesale price of Excavator 1 is an increasing function of social responsibility competition intensity, that is, with the increase in responsibility competition, the wholesale price of Excavator 1 gradually increases. Combined with observations regarding black line in the figure, it appears that when the quantity competition is weak, CSR is not conducive to an increase in the excavator’s wholesale price. When the social responsibility competition intensity is (0, 0.9999), the wholesale price relationship of Excavator 1 under the three models is $w_{1}^{SS}>w_{1}^{NN}>w_{1}^{SN}$. The wholesale price value of Excavator 1 gradually evolves according to *SS→NN→SN* model. When the social responsibility competition intensity is (0.9999, 2.3742), the wholesale price relationship of Excavator 1 under the three models is $w_{1}^{NN}>w_{1}^{SS}>w_{1}^{SN}$, thus showing a gradual evolution according to *NN→SS→SN* model. When the social responsibility competition is (2.3742, 6), the wholesale price relationship of Excavator 1 under the three models is $w_{1}^{NN}>w_{1}^{SN}>w_{1}^{SS}$, and it evolves gradually according to *NN→SN→SS* model.

When the quantity competition intensity is strong (red line in Fig 4), in *SS* model, the wholesale price of Excavator 1 first decreases and then increases with the increase in social responsibility competition intensity. In *SN* model, the wholesale price of Excavator 1 first increases and then decreases with the increase in social responsibility competition intensity, but with the increase of social responsibility competition intensity, the quantity competition is strong. Most of the wholesale prices with a strong degree of competition are larger than those are in a weak degree of quantitative competition, that is, most of the red line is above the black line. Therefore, in the environment of strong competition (more enterprises of the same type), the social responsibility of enterprises is conducive to the increase in the wholesale price of Excavator 1. In other words, when enterprises purchase fewer products, they will not take into account the social responsibility of enterprises when they purchase products. When the social responsibility competition intensity is (0, 1.0001), under the three models, the wholesale price relationship of Excavator 1 is $w_{1}^{SS}>w_{1}^{NN}>w_{1}^{SN}$, and it evolves gradually according to the *SS→NN→SN* model. When the social responsibility competition intensity is (1.0001, 1.6717), the wholesale price relationship of Excavator 1 under the three models is in $w_{1}^{NN}>w_{1}^{SS}>w_{1}^{SN}$, and it evolves gradually according to the *NN→SS→SN* model. When the social responsibility competition intensity is (1.6717, 4.6000), the wholesale price relationship of Excavator 1 under the three models is $w_{1}^{NN}>w_{1}^{SN}>w_{1}^{SS}$, and it evolves gradually according to the *NN→SN→SS* model. When the social responsibility competition intensity is (4.6000, 6), the wholesale price relationship of Excavator 1 under the three models is $w_{1}^{SS}>w_{1}^{NN}>w_{1}^{SN}$, and it evolves gradually according to the mode of *SS→NN→SN*.

**Fig 5:**
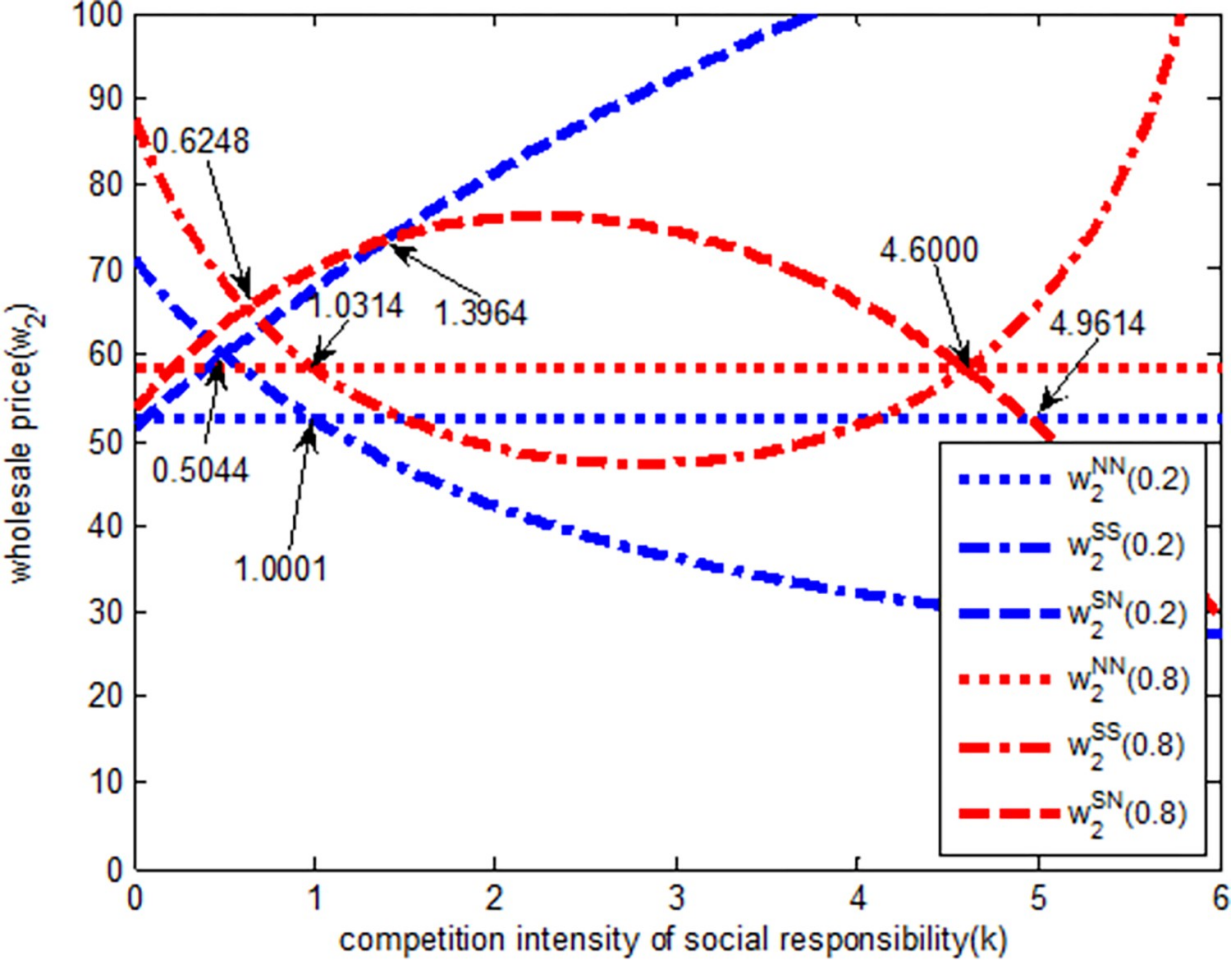
The relationship between wholesale price *w*_*2*_ and completion intensity of social responsibility.

As shown in Fig 5, when the intensity of quantity competition is weak (blue line in the Fig 5), in *SS* model, the wholesale price of Excavator 2 is a decreasing function of social responsibility competition intensity. With the increase in social responsibility competition intensity, the wholesale price of Excavator 2 gradually decreases. In SN model, the wholesale price of Excavator 2 is an increasing function of social responsibility competition intensity, that is, with the increase in responsibility competition, the wholesale price of Excavator 2 gradually increases. Therefore, when the social responsibility competition intensity is high, the SN model is beneficial to the wholesale price increase in excavator 2. When the social responsibility competition intensity is (0, 0.5044), the wholesale price relationship of Excavator 2 under the three models is $w_{2}^{SS}>w_{2}^{SN}>w_{2}^{NN}$. The wholesale price value of Excavator 2 gradually evolves according to *SS→SN→NN* model. When the social responsibility competition intensity is (0.5044, 1.0001), the wholesale price relationship of excavator 2 under the three models is $w_{2}^{SN}>w_{2}^{SS}>w_{2}^{NN}$, and it evolves gradually according to the *SN→SS→NN* model. When the social responsibility competition is (1.0001,6), under the three models, the wholesale price relationship of excavator 2 is $w_{2}^{SN}>w_{2}^{NN}>w_{2}^{SS}$, and it evolves gradually according to *SN→NN→SS* model.

When the quantity competition intensity is strong (red line in the Fig 5), in SS model, the wholesale price of Excavator 2 first decreases and then increases with the increase in social responsibility competition intensity. In SN model, the wholesale price of Excavator 2 first increases and then decreases with the increase in social responsibility competition intensity. Therefore, when the competition intensity of social responsibility is high, the SS model is beneficial to increase the wholesale price of Excavator 2. When the social responsibility competition intensity is (0, 0.6248), the wholesale price relationship of Excavator 2 under the three models is $w_{2}^{SS}>w_{2}^{SN}>w_{2}^{NN}$, and it evolves gradually according to *SS→SN→NN* model. When the social responsibility competition intensity is (0.6248, 1.0314), the wholesale price relationship of Excavator 2 under the three models is $w_{2}^{SN}>w_{2}^{SS}>w_{2}^{NN}$, and it evolves gradually according to the *SN→SS→NN* model. When the social responsibility competition intensity is (1.0314, 4.6000), the wholesale price relationship of Excavator 2 under the three models is $w_{2}^{SN}>w_{2}^{NN}>w_{2}^{SS}$, and it evolves gradually according to the *SN→NN→SS* model. When the competition intensity of social responsibility is (4.6000, 6), the wholesale price relationship of Excavator 2 in the three models is $w_{2}^{SS}>w_{2}^{NN}>w_{2}^{SN}$, and it evolves gradually according to *SS→NN→SN* model.

**Fig 6:**
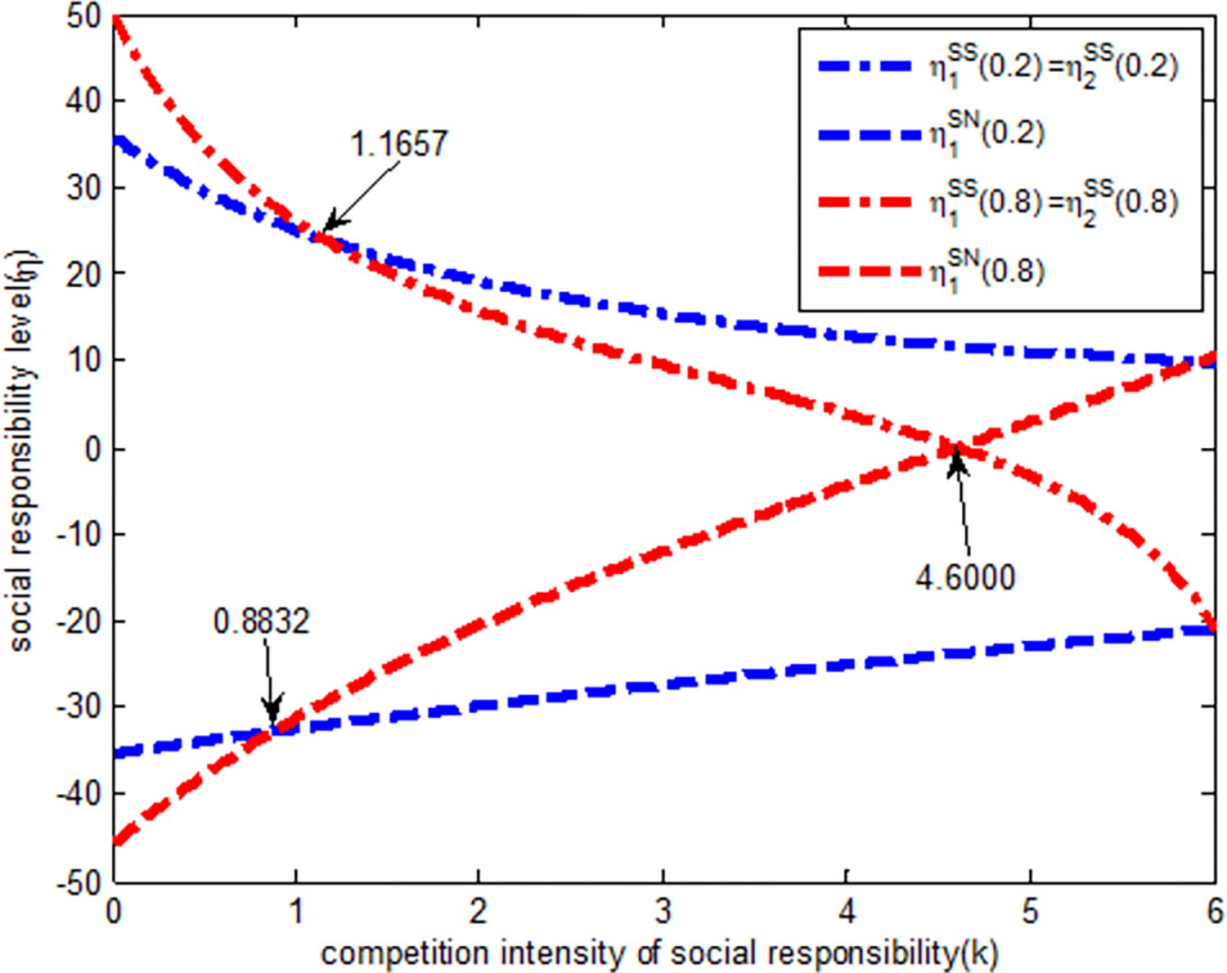
The relationship between social responsibility *η*_1_ and completion intensity of social responsibility.

As shown in Fig 6, regardless of the strength of the quantity competition, in the SS model, the social responsibility level of the excavator is a decreasing function of the social responsibility competition intensity, that is, with an increase in social responsibility competition intensity, the social responsibility level of the excavator gradually decreases. In the SN model, the social responsibility level of the excavator is an increasing function of the social responsibility competition intensity, that is, with an increase in social responsibility competition intensity, the social responsibility level of excavators gradually increases. In the SS model, when the social responsibility competition intensity is (0, 1.1657) and the intensity of quantitative competition is strong, the social responsibility of excavators is always higher than that when the intensity of quantitative competition is weak. When the intensity of social responsibility competition is (1.1657, 6) and the intensity of quantitative competition is weak, the social responsibility level of excavators is always higher than that when the intensity of quantitative competition is strong. If the competition intensity exceeds 4.6000, the level of social responsibility will change from SS model to SN model, that is, the social responsibility level of excavators in SN model is greater than that in SS model.

**Fig 7:**
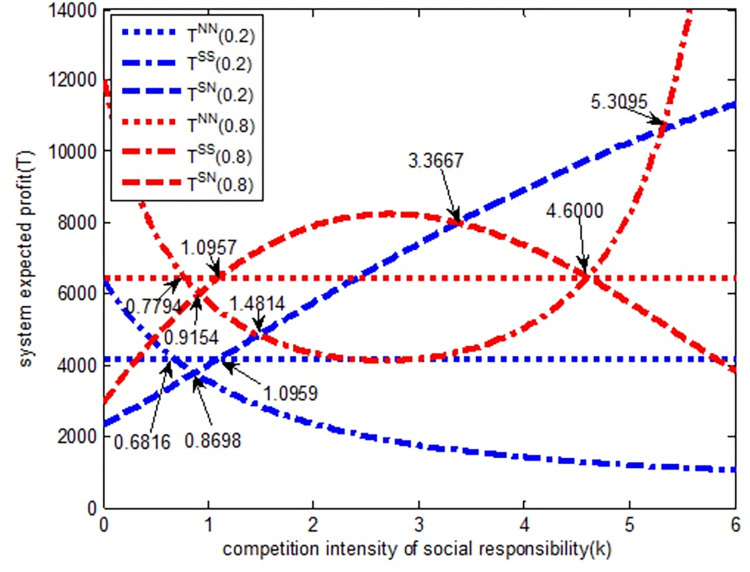
The relationship between systems expected profit and completion intensity of social responsibility.

As shown in Fig 7, regardless of the strength of the quantity competition, in *SS* model, the expected profit of the whole supply chain system is a decreasing function of the social responsibility competition intensity, that is, with an increase in social responsibility competition intensity, the wholesale price of Excavator 2 gradually decreases. In SN model, the expected profit of the whole supply chain system is an increasing function about the social responsibility competition intensity. In other words, with the increase of social responsibility competition intensity, the expected profit of the whole supply chain system gradually increases. Therefore, when the social responsibility competition intensity is very weak (*k*<0.6816), the SS model is beneficial to the expected profit of the whole system; when the social responsibility competition intensity is greater than 1.0959, the SN model is conducive to the increase of the expected profit of the whole supply chain system. When the social responsibility competition intensity is (0, 0.6818), the expected profit relationship of the whole supply chain system under the three models is *T*^*SS*^>*T*^*NN*^>*T*^*SN*^. From the perspective of the expected profit value of the whole supply chain system, it gradually evolves according to *SS→NN→SN* model. When the competition intensity of social responsibility is (0.6818, 0.8698), the expected profit relationship of the whole supply chain system under the three models is *T*^*NN*^>*T*^*SS*^>*T*^*SN*^, and it evolves according to *NN→SS→SN* model. When the social responsibility competition intensity is (0.8698, 1.0959), the expected profit relationship of the whole supply chain system under the three models is *T*^*NN*^>*T*^*SN*^>*T*^*SS*^, and it evolves gradually according to the *NN→SN→SS* model. When the social responsibility competition intensity is (1.0959, 6), the expected profit relationship of the whole supply chain system under the three models is *T*^*SN*^>*T*^*NN*^>*T*^*SS*^, and it evolves gradually according to the *SN→NN→SS* model.

When the quantity competition intensity is strong (red line in the Fig 7), in *SS* model, the expected profit of the whole supply chain system first decreases and then increases with the increase in the social responsibility competition intensity. In SN model, the expected profit of the whole supply chain system first increases and then decreases with the increase of social responsibility competition intensity. Therefore, when the social responsibility competition intensity is very weak (*k*<0.7794) or large (*k*>4.6000), the *SS* model is beneficial to the expected profit of the whole supply chain system. When the social responsibility competition intensity is (0, 0.7794), the expected profit relationship of supply chain system under the three models is *T*^*SS*^>*T*^*NN*^>*T*^*SN*^. From the perspective of the expected profit value of the whole supply chain system, it gradually evolves according to the *SS→NN→SN* model. When the competition intensity of social responsibility is (0.7794, 0.9154), the expected profit relationship of supply chain system under the three models is *T*^*NN*^>*T*^*SS*^>*T*^*SN*^, and it evolves gradually according to the *NN→SS→SN* model. When the competition intensity of social responsibility is (0.9154, 1.0957), the expected profit relationship of supply chain system under the three models is *T*^*NN*^>*T*^*SN*^>*T*^*SS*^, and it evolves gradually according to the *NN→SN→SS* model. When the social responsibility competition intensity is (1.0957, 4.6000), the expected profit relationship of supply chain system under the three models is *T*^*SN*^>*T*^*NN*^>*T*^*SS*^, and it evolves gradually according to the *SN→NN→SS* model. When the competition intensity of social responsibility is in (4.6000,6), the expected profit relationship of supply chain system under the three models is *T*^*SS*^>*T*^*NN*^>*T*^*SN*^ and it evolves gradually according to the *SS→NN→SN* model.

No matter how the quantity competition intensity changes, when the social responsibility competition intensity is (0, 3.3667) and (5.3095, 6), the expected profit of the supply chain system is always greater than the supply chain system without social responsibility. This further shows that corporate social responsibility behavior promotes the overall performance of the supply chain. The government can take some policies on social responsibility, adjust the competition intensity of social responsibility timely, make the enterprises in the market pay attention to their own social responsibility, form a good social atmosphere, which is conducive to the stable and sustainable development of economy.

## 7 Conclusion and policy implications

With the increasing proportion of personalized consumers in the market, enterprises will pay increasingly more attention to whether their products can recognized by consumers, especially in the new epidemic situation. People pay more and more attention to product safety, which also makes them pay increased attention to the social responsibility of enterprises. In this paper, we consider a competitive mining supply chain system consisting of two excavators (M1 and M2) and two exclusive retailers (R1 and R2). The excavator can have a certain sense of social responsibility, that is, in addition to pursuing economic profits, they can also consciously pay attention to the environmental and health concerns of the local people (e.g., green production of better products). We have established a game model of social responsibility for three different subjects. Two excavators do not show CSR behavior (NN). Two excavators show CSR behavior (SS). One excavator shows CSR behaviour (SN). Through the optimization theory, we solved the optimal strategy of the mineral supply chain, and analysed how parameter changes affect the strategy and member performance. The results show that: first, the optimal decisions of the mining supply chain members are the same in each case under the *NN* and *SS* models. In the *SN* model, the optimal decision strategy value of mining supply chain members is always greater than non-socially responsible supply chain members. Second, excavators in both chains engage in CSR, the level of CSR increases with the increase in CSR competition. When the social responsibility competition intensity is small, the excavator’s wholesale price and retailer’s order price are both the decreasing function of the social responsibility competition intensity. When the social responsibility competition intensity is large, the excavator’s wholesale price and the retailer’s order price are both increasing functions of the social responsibility competition intensity. Third, when only one excavator in the two chains exhibits social responsibility, and the social responsibility level of the excavators increases with the increase in the social responsibility competition intensity. When the competition intensity of social responsibility is small, the wholesale price of excavators and the order price of retailers in the mining supply chain are both increasing functions of the intensity of social responsibility competition. The wholesale price of the excavator and the order price of the retailer in the supply chain where CSR exhibited are both the decreasing functions of the competitive intensity of social responsibility.

To further encourage and guide mining enterprises to consider environmental protections, people’s health, and other social responsibility issues related to production, customers and the government can advocate for and provide policy measures from the following two aspects:

Firstly, when purchasing goods, customers can choose mineral enterprises who show a strong sense of social responsibility. Compared with mineral enterprises without a sense of social responsibility, the order price is higher, but the product quality of socially responsible enterprises is better and the pollution is less. If the government can formulate a series of price compensation mechanisms to compensate customers who prefer social responsibility, this will help improve corporate social responsibility awareness, and thus change the overall sense of social responsibility.

Secondly, the government can adopt a flexible price mechanism to control the output of mineral enterprises. We encourage mineral enterprises with a strong sense of social responsibility to produce more products and cause less pollution to society. We can also give some financial support to mineral enterprises with weak social responsibility, and encourage them to improve their production processes and processes, so as to improve their social responsibility.

This paper has several limitations. Firstly, it is assume that the members of the supply chain are neutral to risk. The risk attitude of supply chain members should considered as the different risk attitudes of supply chain members will inevitably lead to different operational decisions and thereby affect the operation decisions of the downstream or upstream supply chain members. Therefore, future studies can evaluate the competitive enterprises with social responsibility behaviour in the case of risk-aversion business supply chain decision-making. Moreover, the outbreak of the new coronavirus epidemic has caused some enterprises to face financial constraints, which leads to a scientific problem. Given the limited funds, how can enterprises share the production funds and social responsibility cost input to ensure the normal operations and profits of enterprises? What kind of financing methods will enterprises employ to break the capital bottleneck and solve the capital constraints? These are the problems in require further study.

## Supporting information

S1 Appendix (DOCX)

S1 File The calculation method of basic data. (DOCX)
