# Supplementary material for: Operation decision of competitive mining supply chain based on social responsibility
Source: PLoS One. 2022 Dec 8;17(12):e0278815. doi: 10.1371/journal.pone.0278815 (PMC9731416; doi:10.1371/journal.pone.0278815)
Supplement: S1 Appendix — (DOCX) [file pone.0278815.s001.docx]

**Appendix A.**

***Proof of proposition 1:***

In this case, because the excavators do not fulfill their social responsibility behavior, we can follow the general competitive supply chain model. According to the expressions (3) and (4), and using the inverse induction method, we can get that,

$w_{i}^{NN*}=\frac{a\left(2\text{+}b\right)}{b+4}$ (A1)

$q_{i}^{NN*}=\frac{2a}{\left(b+4\right)\left(2-b\right)}$ (A2)

Proposition 1 is proved.

***Proof of proposition 2:***

In this case, the excavators have assumed social responsibility. According to the solution of proposition 1, we can get,

$w_{i}^{SS*}=\frac{a\left(16-b^{2}\right)\left(4-b^{2}\right)}{-8bk^{2}-4\left(b^{2}-2b-8\right)k+b^{4}+2b^{3}-20b^{2}-32b+96}$ (A3)

$\eta_{i}^{SS*}=\frac{4a\left(8-2bk-b^{2}\right)}{-8bk^{2}-4\left(b^{2}-2b-8\right)k+b^{4}+2b^{3}-20b^{2}-32b+96}$ (A4)

$q_{i}^{SS*}=\frac{2a\left(16-b^{2}\right)}{-8bk^{2}-4\left(b^{2}-2b-8\right)k+b^{4}+2b^{3}-20b^{2}-32b+96}$ (A5)

Proposition 2 is proved.

***Proof of proposition 3:***

In this case, because the M1 fulfill its social responsibility and the M2 don’t fulfill its social responsibility behavior. According to the solution of proposition 1, we can get that,

$w_{1}^{SN*}=\frac{a\left(2+b\right)\left(b^{6}-28b^{4}+256b^{2}-512+32b^{2}k^{2}+32b^{3}k-256bk\right)}{\left(4+b\right)\left[16b^{2}k^{2}-16b\left(b^{2}-8\right)k+b^{6}-32b^{4}+320b^{2}-768\right]}$ (A6)

$w_{2}^{SN*}=\frac{a\left(4-b\right)\left(2+b\right)^{2}\left[-b^{4}-2b^{3}+20b^{2}+32b-96+4\left(b^{2}-2b-8\right)k+8bk^{2}\right]}{\left(4+b\right)\left[16b^{2}k^{2}-16b\left(b^{2}-8\right)k+b^{6}-32b^{4}+320b^{2}-768\right]}$ (A7)

$\eta_{1}^{SN*}=\frac{4a\left(2+b\right)\left(4-b\right)\left(b^{2}+2bk-8\right)}{-16b^{2}k^{2}+16b\left(b^{2}-8\right)k-b^{6}+32b^{4}-320b^{2}+768}$ (A8)

$q_{1}^{SN*}=\frac{2a\left(b^{6}-28b^{4}+256b^{2}-512+32b^{2}k^{2}+32b^{3}k-256bk\right)}{\left(2-b\right)\left(4+b\right)\left[16b^{2}k^{2}-16b\left(b^{2}-8\right)k+b^{6}-32b^{4}+320b^{2}-768\right]}$ (A9)

$q_{2}^{SN*}=\frac{2a\left(4-b\right)\left(2+b\right)\left[-b^{4}-2b^{3}+20b^{2}+32b-96+4\left(b^{2}-2b-8\right)k+8bk^{2}\right]}{\left(2-b\right)\left(4+b\right)\left[16b^{2}k^{2}-16b\left(b^{2}-8\right)k+b^{6}-32b^{4}+320b^{2}-768\right]}$ (A10)

Proposition 3 is proved.

***Proof of proposition 4:***

We find the first derivatives of (A5) and (A3) with respect to *k*, then

$\frac{\partial q_{i}^{SS}}{\partial k}\text{=}\frac{-2a\left(16-b^{2}\right)\left(32+8b-16kb-4b^{2}\right)}{\left[96-32b-20b^{2}+2b^{3}+b^{4}-\left(4b^{2}-8b-32\right)k-8bk^{2}\right]^{2}}$,

$$\frac{\partial w_{i}^{SS}}{\partial k}\text{=}\frac{-a\left(4-b^{2}\right)\left(16-b^{2}\right)\left(32+8b-16kb-4b^{2}\right)}{\left[96-32b-20b^{2}+2b^{3}+b^{4}-\left(4b^{2}-8b-32\right)k-8bk^{2}\right]^{2}}$$

In order to judge the sign of$\frac{\partial q_{i}^{SS}}{\partial k}$, we only need to look at the sign of $32+8b-16kb-4b^{2}$. We make$32+8b-16kb-4b^{2}\text{=}0$, then$k\text{=}\frac{\left(4+b\right)\left(2-b\right)}{4b}$. According to the properties of the function, when $k>\frac{\left(4+b\right)\left(2-b\right)}{4b}$, then $32+8b-16kb-4b^{2}\text{<}0$, and $\frac{\partial q_{i}^{SS}}{\partial k}>0$,$\frac{\partial w_{i}^{SS}}{\partial k}>0$; when $0<k<\frac{\left(4+b\right)\left(2-b\right)}{4b}$, then $32+8b-16kb-4b^{2}>0$, and $\frac{\partial q_{i}^{SS}}{\partial k}<0$, $\frac{\partial w_{i}^{SS}}{\partial k}<0$.

Proposition 4 is proved.

***Proof of proposition 5:***

We find the first derivative of (A4) with respect to *k*, then

$\frac{\partial\eta_{i}^{SS}}{\partial k}\text{=}\frac{-8a\left(128+128b-64b^{2}-24b^{3}+4b^{4}+b^{5}-64kb+8kb^{3}+8k^{2}b^{2}\right)}{\left[96-32b-20b^{2}+2b^{3}+b^{4}-\left(4b^{2}-8b-32\right)k-8bk^{2}\right]^{2}}$,

In order to judge the sign of$\frac{\partial\eta_{i}^{SS}}{\partial k}$, we only need to look at the sign of $128+128b-64b^{2}-24b^{3}+4b^{4}+b^{5}-64kb+8kb^{3}+8k^{2}b^{2}$. We find that its value is always greater than zero. When$k>0$, then $\frac{\partial\eta_{i}^{SS}}{\partial k}<0$.

Proposition 5 is proved.

***Proof of proposition 6:***

We find the first derivatives of (A9) and (A6) with respect to *k*, then

$\frac{\partial q_{1}^{SN}}{\partial k}\text{=}\frac{-32ab\left(2kb+b^{2}-8\right)\left(b+4\right)\left(b+2\right)\left(4-b\right)^{2}}{\left(-768-128kb+16b^{2}k^{2}+320b^{2}+16kb^{3}-32b^{4}+b^{6}\right)^{2}}$,

$$\frac{\partial w_{1}^{SN}}{\partial k}\text{=}\frac{-16ab\left(2kb+b^{2}-8\right)\left(b+4\right)\left(2-b\right)\left(2+b\right)^{2}\left(4-b\right)^{2}}{\left(-768-128kb+16b^{2}k^{2}+320b^{2}+16kb^{3}-32b^{4}+b^{6}\right)^{2}}$$

In order to judge the sign of$\frac{\partial q_{1}^{SN}}{\partial k}$, we only need to look at the sign of $2kb+b^{2}-8$. We make $2kb+b^{2}-8\text{=}0$, then $k\text{=}\frac{\left(8-b^{2}\right)}{2b}$. According to the properties of the function, when$k>\frac{\left(8-b^{2}\right)}{2b}$, then $2kb+b^{2}-8>0$, and $\frac{\partial q_{1}^{SN}}{\partial k}<0$, $\frac{\partial w_{1}^{SN}}{\partial k}<0$.When $0<k<\frac{\left(8-b^{2}\right)}{2b}$, then $2kb+b^{2}-8<0$, and $\frac{\partial q_{1}^{SN}}{\partial k}>0$, $\frac{\partial w_{1}^{SN}}{\partial k}>0$.

Proposition 6 is proved.

***Proof of proposition 7:***

We find the first derivatives of (A10) and (A7) with respect to *k*, then

$\frac{\partial q_{2}^{SN}}{\partial k}\text{=}\frac{-8a\left(b+2\right)\left(4-b\right)\left(b^{6}+4kb^{5}-24b^{4}-80kb^{3}+16k^{2}b^{2}+384kb+192b^{2}-768\right)}{\left(-768-128kb+16b^{2}k^{2}+320b^{2}+16kb^{3}-32b^{4}+b^{6}\right)^{2}}$,

$$\frac{\partial w_{2}^{SN}}{\partial k}\text{=}\frac{-4a\left(2-b\right)\left(4-b\right)\left(2+b\right)^{2}\left(b^{6}+4kb^{5}-24b^{4}-80kb^{3}+16k^{2}b^{2}+384kb+192b^{2}-768\right)}{\left(-768-128kb+16b^{2}k^{2}+320b^{2}+16kb^{3}-32b^{4}+b^{6}\right)^{2}}$$

In order to judge the sign of$\frac{\partial q_{2}^{SN}}{\partial k}$, we only need to look at the sign of $b^{6}+4kb^{5}-24b^{4}-80kb^{3}+16k^{2}b^{2}+384kb+192b^{2}-768$. We make $b^{6}+4kb^{5}-24b^{4}-80kb^{3}+16k^{2}b^{2}+384kb+192b^{2}-768\text{=}0$, then $k\text{=}\frac{-b^{5}+20b^{3}-96b+\sqrt{H_{1}}}{8b}$. Where $H_{1}\text{=}12288-4608b^{2}+688b^{4}-44b^{6}+b^{8}$. According to the properties of the function, when$k>\frac{-b^{5}+20b^{3}-96b+\sqrt{H_{1}}}{8b}$, then $b^{6}+4kb^{5}-24b^{4}-80kb^{3}+16k^{2}b^{2}+384kb+192b^{2}-768\text{>}0$, and $\frac{\partial q_{2}^{SN}}{\partial k}<0$,$\frac{\partial w_{2}^{SN}}{\partial k}<0$. When$0<k<\frac{-b^{5}+20b^{3}-96b+\sqrt{H_{1}}}{8b}$, then $b^{6}+4kb^{5}-24b^{4}-80kb^{3}+16k^{2}b^{2}+384kb+192b^{2}-768<0$, and $\frac{\partial q_{2}^{SN}}{\partial k}>0$,$\frac{\partial w_{2}^{SN}}{\partial k}>0$.

Proposition 7 is proved.

***Proof of proposition 8:***

We find the first derivatives of (A8) with respect to *k*, then

$$\frac{\partial\eta_{1}^{SN}}{\partial k}\text{=}\frac{8ab\left(4-b\right)\left(1280+16k^{2}b^{2}+16kb^{3}-128kb+40b^{4}-b^{6}-448b^{2}\right)}{\left(-768-128kb+16b^{2}k^{2}+320b^{2}+16kb^{3}-32b^{4}+b^{6}\right)^{2}}$$

In order to judge the sign of $\frac{\partial\eta_{1}^{SN}}{\partial k}$, we only need to look at the sign of $1280+16k^{2}b^{2}+16kb^{3}-128kb+40b^{4}-b^{6}-448b^{2}$. We find that its value is always greater than zero. When$k>0$, then $\frac{\partial\eta_{1}^{SN}}{\partial k}>0$.

Proposition 8 is proved.
