# Supplementary material for: Operation decision of competitive mining supply chain based on social responsibility
Source: PLoS One. 2022 Dec 8;17(12):e0278815. doi: 10.1371/journal.pone.0278815 (PMC9731416; doi:10.1371/journal.pone.0278815)
Supplement: S1 File — (DOCX) [file pone.0278815.s002.docx]

The calculation method of basic data

We assume a=100, b=0.2, b=0.5 and b=0.8.

In Fig.2, a=100, b=0.2. Combined with the formula A2,A5,A9 in the Appendix A. Take a=100, b=0.2 into the formula A2,A5,A9, we can get the relationship between the order quantity and k under three scenarios.

Other figures are available in the same way. Thank you.
